# Retraction: Role of DNA Methylation in Cell Cycle Arrest Induced by Cr (VI) in Two Cell Lines

**DOI:** 10.1371/journal.pone.0333845

**Published:** 2025-10-07

**Authors:** The PLOS One Editors

After this article [1] was published, concerns were raised regarding results presented in Fig 3.

Specifically:

- Fig 3A lanes 1-5 appear similar to Fig 3B lanes 1-5
- There are multiple regions in the background that appear discontinuous with adjacent background regions in the following panels:
  - Fig 3A
  - Fig 3B
  - Fig 3C
  - Fig 3D
- There appear to be one or more vertical discontinuities between lanes in the following panels:
  - Fig 3A
  - Fig 3C
  - Fig 3D

Co-corresponding author JL stated that Figs 3A-D were spliced from multiple gels as there were 18 lanes in the original gels and 21 lanes in each of Figs 3A-D, and that in Fig 3D the bands in lanes 6 and 8 were lowered relative to the other bands from the same underlying blot. They stated that lanes 2–5 in Fig 3A are the same as lanes 2–5 in Fig 3B as the same positive and negative controls were used in these figures. They provided some of the underlying original gel data for Figs 3A-3D, but stated that not all original data could be located. Upon editorial review, these underlying data were insufficient to resolve the above concerns, and raised further concerns for the reliability and validity of the published results.

In light of the above concerns, the *PLOS One* Editors retract this article.

JL agreed with the retraction, stands by the article’s findings, and apologizes for the issues with the published article. YW, CY, LJ, XW, YX, NW, PS, YS, YT, MG, KL, and XZ either did not respond directly or could not be reached.
